# CMOS Time-Resolved, Contact, and Multispectral Fluorescence Imaging for DNA Molecular Diagnostics

**DOI:** 10.3390/s141120602

**Published:** 2014-10-31

**Authors:** Nan Guo, Ka Wai Cheung, Hiu Tung Wong, Derek Ho

**Affiliations:** Department of Physics and Materials Science, City University of Hong Kong, 83 Tat Chee Avenue, Kowloon, Hong Kong; E-Mails: nanguo-c@my.cityu.edu.hk (N.G.); kwcheun44-c@my.cityu.edu.hk (K.C.); htwong39-c@my.cityu.edu.hk (H.T.W.)

**Keywords:** CMOS, FRET, lab-on-a-chip, microsystem, fluorescence spectroscopy, multispectral imaging, contact imaging, quantum dots, DNA analysis, point-of-care, medical diagnostics, time-resolved, TCSPC

## Abstract

Instrumental limitations such as bulkiness and high cost prevent the fluorescence technique from becoming ubiquitous for point-of-care deoxyribonucleic acid (DNA) detection and other in-field molecular diagnostics applications. The complimentary metal-oxide-semiconductor (CMOS) technology, as benefited from process scaling, provides several advanced capabilities such as high integration density, high-resolution signal processing, and low power consumption, enabling sensitive, integrated, and low-cost fluorescence analytical platforms. In this paper, CMOS time-resolved, contact, and multispectral imaging are reviewed. Recently reported CMOS fluorescence analysis microsystem prototypes are surveyed to highlight the present state of the art.

## 1. Introduction

Deoxyribonucleic acid (DNA) analysis platforms are used in the life sciences for the observation, identification, and characterization of various biological systems. These platforms serve applications such as pathogen detection [1,2], disease screening [3,4], biohazard detection [5,6], cancer diagnostics [7,8], and genetic research [9–11]. Biosensors are a subset of such platforms that can convey biological parameters in terms of electrical signals. Biosensors measure the quantity of various biological analytes and are often required to be capable of specifically detecting multiple analytes simultaneously. A goal in biosensor research is to develop portable, hand-held devices for point-of-care (POC) use, for example, in a physician's office, an ambulance, or at a hospital bedside that can provide time-critical information about a patient at the location of need [1].

The current demand for high-throughput, point-of-care bio-recognition has introduced new technical challenges for biosensor design and implementation. Conventional biological tests are highly repetitive, labor intensive, and require a large sample volume [9,12]. The associated biochemical protocols often require hours or days to perform at a cost of hundreds of dollars per test. Instrumentation for performing such testing today is bulky, expensive, and requires considerable power consumption. Problems remain in detecting and quantifying low levels of biological compounds reliably, conveniently, safely, and quickly. Solving these problems require the development of new assay and sensor techniques.

Well-known molecular sensing techniques include electrochemical detection [3,13], surface plasmon resonance [14], and fluorescence [2,4,5]. In electrochemical DNA detection, for example, a charge-transfer chemical reaction causes a change in the electrical properties of the system. Subcategories of electrochemical methods include cyclic voltammetry, constant potential amperometry, and impedance spectroscopy [3,13]. Detection may be label-free or requires labeling. In the label-free case, the presence of the target causes an observable signal without requiring the attachment of a label molecule to the sample [15–17]. To improve selectivity, labels are used to enhance the sensor response to particular targets. The electrochemical system typically involves three electrodes (*i.e*., reference, working, and working electrodes). Single-stranded DNA probes are first immobilized on the electrodes and then immersed in an electrolyte solution. Next, single-stranded DNA targets that have been labeled with an electroactive species are introduced to the electrolyte and allowed to interact with the probes. These labels are designed to transfer charge to the electrode when a potential is applied. Then, a potential is applied between the two electrodes and only labels attached to surface-hybridized targets are able to transfer charge to the electrode. A quantitative measure of the degree of hybridization, reflecting the target concentration, is obtained by monitoring the reduction-oxidation current. Although electrochemical techniques are capable of highly spatially multiplexed detection and often leads to simple electronic readout, it is generally more difficult to achieve combined high selectivity and high sensitivity as compared to an assay using well-chosen optical labels to interrogate specific processes [3,5,13].

Surface plasmons are collective oscillation of electrons in the conduction band of a thin conductive metal film. Surface plasmon resonance [14] refers to optical excitation of surface plasmons, *i.e.*, when the electric field of an incoming radiation (such as a laser source) is in resonance with the electric field of surface plasmons to stimulate excitation of surface plasmons. The hybridization of a target strand to a probe strand that is immobilized in close proximity to the thin metal film results in a change in the optical mass (refractive index and/or mass), which changes the resonance conditions for excitation of surface plasmons. This change is transduced in terms of a change in the incidence angle for maximal light absorption, which serves as an analytical signal. An advantage of using surface plasmon resonance for optical interrogation of nucleic acid detection is that it is a label-free technique. However, this technique is not easily applicable to an array sensor implementation, which limits the overall sensor throughput.

Fluorescence-based detection has arguably become the standard amongst many analytical methods [9]. To illustrate the principle of fluorescence detection, consider the optically-labeled DNA microarray. Microarrays enable highly-multiplexed and parallel detection. Typically, single-stranded DNA (ssDNA) probe molecules are arranged in a regular pattern on a passive substrate, such as a glass slide. Probes are then allowed to hybridize with complementary, fluorophore-labeled ssDNA target molecules. After that, non-hybridized targets, *i.e.*, those not sought after, are removed from the array through a washing step. The light from the labels attached to the hybridized targets is then detected by an instrument. By choosing from a wealth of optical labels available such as fluorophores and quantum dots (QDs), the fluorescence technique is capable of simultaneously achieving highly sensitive and selectivity analysis [12,18,19].

Instrument used for fluorescence detection are typically bulky and expensive, which render them unsuitable for portable, point-of-care applications [10]. This calls for the reduction of sensor complexity, size, and cost, which can be achieved using the complementary metal-oxide-semiconductor (CMOS) technology. CMOS offers many advantages such as low cost, high integration density, and signal processing versatility [4,6,8,20].

In this paper, fluorescence detection techniques that maps well to CMOS sensor implementation are reviewed. Three techniques, specifically time-resolved, contact, and multispectral imaging are discussed, including a survey of state of the art CMOS realizations. The rest of the paper is organized as follows. Section 2 provides the background information of fluorescence spectroscopy such as operating principle and performance metrics. Section 3 discusses drawbacks of existing fluorescence detection technologies and motivates for solutions. Section 4 describes CMOS techniques that mitigate the drawbacks and surveys recently reported implementations. Section 5 concludes the paper.

## 2. Background

### 2.1. Principle of Operation

**Figure 1.**
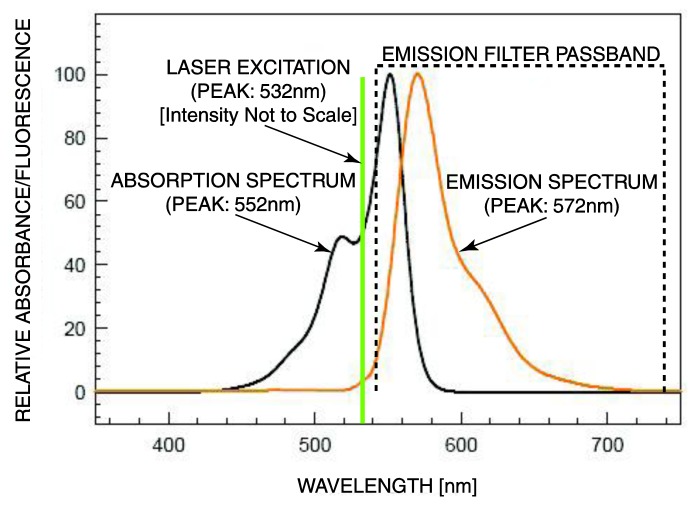
Key spectra of the commonly used Cyanine3 (Cy3) fluorescent molecule.

In the standard form of laser-induced fluorescence [9,11], a fluorescent molecule, also known as a fluorescent label or a fluorophore, is attached to each of the target molecules through a labeling step. The fluorophore, upon absorbing photons at one wavelength band, emits photons at a band of a longer wavelength. Fluorophores such as Cy3, Cy5, and fluorescein are commonly used as fluorescent molecules and usually emit light with the wavelengths in the 500 nm to 700 nm range. In addition, they suffer from photobleaching, which limits the duration of excitation applied to the sample. Figure 1, adopted from [10], depicts the absorption and emission spectra of the Cy3 fluorophore. Multiple fluorophores, e.g., green and red labels, are sometimes used to detect multiple targets. Upon excitation, light is given off if a molecular target is found. The light is collected typically by a photodetector after passing through an optical filter that rejects the excitation.

Aside from well-adopted fluorophores such as organic dyes [9], a class of colloidal semiconductor nanocrystals, known as QDs [21], exhibit a number of unique optical properties that are advantageous to be used as labels, especially for spectral multiplexing. These unique properties include: narrow, symmetric and size-tunable emission photoluminescence spectra (full width at half maximum, FWHM, 25–35 nm); strong and broad absorption spectra; high quantum yields (> 20%) and long life times (> 10 ns) [19]. As compared to organic fluorophores, QDs have greater resistance to photobleaching that enables long-term monitoring. The broad absorption spectra of QDs allows for multiple colors of QDs to be excited efficiently with a single excitation source which is not possible with organic dyes. These optical properties of QDs make them suitable for optical multiplexing and as donors for Förster resonance energy transfer (FRET) applications. FRET is a mechanism that transfers energy between two molecules through space dipolar coupling. A donor molecule may transfer energy to an acceptor molecule, over a distance typically less than 10 nm, through non-radiative dipole-dipole coupling [22].

### 2.2. Sensor Performance Metrics

Important measures of performance for DNA and molecular sensors include detection limit, selectivity, and dynamic range [3,13]. The detection limit is the lowest amount of the target that can be reliably detected by the sensor, or in the case of a solution, the lowest target concentration that can be detected. In practice, a specific signal-to-noise ratio (SNR) is often used to define the detection limit, e.g., an SNR of 3 dB. The noise level can be obtained from the sensor response to a buffer solution.

Selectivity refers to the ability of a sensor to respond only to the desirable target in a sample containing other molecules. For nucleic acid detection, selectivity is often governed by the environment of hybridization such as ionic strength of solution and temperature.

The dynamic range of the sensor is the ratio of the highest to the lowest target concentrations that result in a predictable (linear or logarithmic) response from the sensor. The former parameter is often limited, in the chemistry, by the maximum amount of target molecules that can hybridize onto the probe layer (due to finite probe surface area) or, in the electronics domain, by the saturation level of the detector.

## 3. Challenges and Recent Progress in Fluorescence Detection

Current challenges for fluorescence detection are strong background signal, bulky equipment, and high spatial variability. They are discussed below, with possible alternatives to overcome the challenges.

### 3.1. Strong Background Signal

#### 3.1.1. Conventional Approach: Continuous Excitation, Exposure-Based Detection

Many fluorescent dyes such as fluorescein, rhodamine and cyanine are well known as markers to signal the hybridization of nucleic acids. However, there is a main problem of eliminating background noises caused by autofluorescence from biological samples, the scattering light from solid substrates, and the luminescence from the optical components [23]. Conventional photo detection involves the integration of the photocurrent when the excitation operates continuously in the background. This makes background suppression very difficult, especially in a microsystem where optical filter performance is often traded off for small size.

#### 3.1.2. Alternative: Pulsed Excitation, Time-Resolved Detection

**Figure 2.**
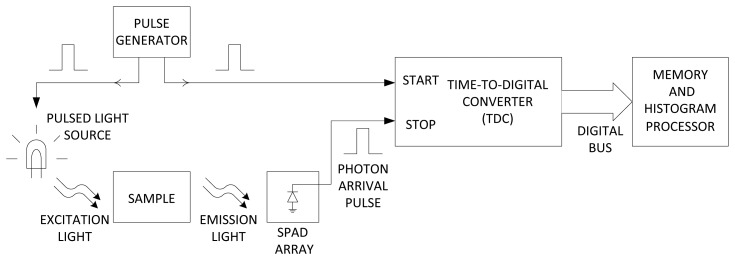
Time-correlated single photon counting (TCSPC) system block diagram.

Time-resolved measurement is an effective way to mitigate the problem with a large background signal, for example due to autofluorescence. Time-resolved imaging is a technique where low-light signals are recorded with high timing resolution relative to a synchronized optical impulse excitation, in order to extract the characteristic luminescence decay constant or lifetime. When fluorophores are excited by a short pulse of light, the fluorescence signal decays exponentially. The decay constant depends on the surrounding of the fluorophore, for example, the molecules in its surroundings. Therefore, by measuring the decay profile, or lifetime, molecular sensing can be achieved. For example in time-correlated single photon counting (TCSPC), upon each excitation pulse, the emitted fluorescence photons are detected. The arrival time of photons are recorded in a histogram. A typical TCSPC apparatus includes a pulsed light source (typically a laser), a discrete detector such as the photomultiplier tube (PMT), a stand-alone time-to-digital converter (TDC) module, and a workstation to compute the decay constant, as depicted in Figure 2. Since the pulsed laser is turned off during fluorescence detection, the background signal is substantially reduced. However, conventional hardware for photon counting results in bulky, expensive and power-hungry acquisition systems.

Furthermore, major limitation of conventional time-resolved equipment is the restrictively low photon count limit that is below 5% of the excitation rate, which is necessary to avoid distortion due to photon pile-up caused by both long detector dead-time and the inability of the TDC to process more than one event per excitation period. Therefore, TCSPC has in the past been limited by peak acquisition rates of around 1 MHz. For measurements requiring the capture of many samples to achieve a high accuracy, limited acquisition rate can lead to long measurement time, sometimes in the order of hours.

Solid-state single photon detectors such as an avalanche photodiode (APD) and single photon avalanche diode (SPAD) have recently taken on a significant role in the development of high-performance single photon imaging systems. The main driving factors are the unparalleled levels of miniaturization and portability, low fabrication costs, and high overall performance resulting from the integration of SPADs with mixed-signal circuits in deep-submicron (DSM) CMOS technology. At the heart of such imaging systems is the SPAD, capable of single-photon sensitivity and sub-nanosecond time resolution, and its associated circuitry, which in highly-scaled CMOS, is capable of high-speed, low-power mixed-mode signal processing.

### 3.2. Bulky Equipment

#### 3.2.1. Conventional Approach: Heavy Use of Optics

In terms of the instrument size, the multiple lasers and optical detectors employed in the microarray scanners and the widely-used fluorescent microscope render them bulky, which limits portability of these instrumentations for point-of-care applications [10]. In a fluorescent microscope, excitation is typically provided by a laser source, which passes through an excitation filter to remove stray outputs that overlap with the emission wavelengths. Then, the dichroic mirror reflects and directs the excitation to the sample. The fluorophores in the sample absorb the excitation light and emit light at a longer wavelength, which is passed through the dichroic mirror. The emitted light is then directed through an emission filter (for choosing the exact wavelength in a multi-wavelength emission setup), a focusing lens, an aperture (to control the output intensity to prevent detector saturation), and finally reaches the detector, typically a PMT or a cooled charge-coupled device (CCD). PMTs are amongst the most sensitive photo detectors, but are bulky, expensive and require high operational voltage making them unattractive to be integrated into a portable system. The throughput of PMT based detection systems is relatively low due to the lack of parallelism. In contrast, CCDs can be implemented into an array, but do not allow for on-chip integration of peripheral circuits. Implementing signal conditioning circuits on a separate die increases cost and limits miniaturization.

#### 3.2.2. Alternative: Contact Imaging

Innovation in imaging techniques such as contact imaging [10,24,25] can be employed to reduce the size of the conventional bulky and expensive optical detection instruments. Unlike the conventional fluorescent microscope, in contact imaging, as depicted in Figure 2, the object to be imaged is placed in close proximity to the focal plane. In this technique, the resolution of the imaging system is on the order of the pixel size. This technique eliminates the need for bulky and expensive optics such as a system of lenses and mirrors, which enables miniaturization to realize lab-on-a-chip platforms [26–28].

### 3.3. High Variability

#### 3.3.1. Conventional Approach: Spatial Multiplexing

Although the conventional microarray technology employs spatial registration to achieve unprecedented parallelism, it suffers from disadvantages such as additional processing required to print spots on a surface and the associated spatial variation in terms of the quality of the probe immobilization across discrete spots [9,29]. In addition, microarray manufacturing typically involves time-consuming processes and a laboratory environment. They often offer much greater capabilities, such as parallelism, than required for many in-field applications such as pathogen detection [11].

#### 3.3.2. Alternative: Spectral Multiplexing

An alternative to spatial registration is spectral multiplexing. For example, in spectrally-multiplexed fluorescence-based DNA detection, target analytes can be labeled with different fluorophores that can be distinguished by their emission wavelengths. By measuring the emission intensity at each of these wavelengths, different targets, such as nucleic acid targets, can be simultaneously quantified [21]. Unlike other spectroscopic techniques, such as Raman spectroscopy, where continuous fine spectral resolution is required, fluorescent imaging requires spectral differentiation among only a few discrete wavelengths [20,24]. Spectral multiplexing has been successfully demonstrated in several applications.

In [30], for the subcellular measurement of proteins in tissue and cells, the targets of interest are present in the same or spatially overlapping cellular compartments. Such co-localization would complicate analysis and interpretation of the images obtained using traditional ‘single-color’ fluorescence sensors. However, using a color sensor to differentiate among emission bands and filtering them from background autofluorescence, the sensor reported images three targets in a single channel. This parallel imaging approach provides significant advantages for multiplexed analysis of tissues and cells.

Advanced techniques such as FRET can also be realized on a spectrally-multiplexed assay. In [31], a nanoscale sensor employing FRET interactions between fluorescent QDs and organic quencher molecules is used for the multiplexed detection of biological antigens in solution. Detection occurs when the antigens to be detected displace quencher-labelled inactivated (or dead) antigens of the same type attached to QD antibody complexes through equilibrium reactions. This unquenches the QDs, allowing detection to take place through the observation of photoluminescence in solution or through the fluorescence imaging of unquenched QD complexes trapped on filter surfaces. Multiplexing can be accomplished by using several different sizes of QDs, with each size QD labelled with an antibody for a different antigen, providing the ability to detect several types of antigens or biological contaminants simultaneously in near real-time with high specificity and sensitivity.

To interrogate the probe-target hybridization event in DNA analysis, the assay in [2] employs QDs as energy donors and targets labeled with black hole quenchers (BHQs) as acceptors in the FRET process. Green-emitting QDs (gQDs) and red-emitting QDs (rQDs) are conjugated with the survival motor neuron 1 (SMN1) and uidA probes, respectively. The SMN1 sequence is a diagnostic of a neuro-degenerative disease called spinal muscular atrophy. The uidA sequence is a marker for E. coli bacteria. SMN1 targets are labeled with BHQ1 and uidA targets are labeled with BHQ2. Hence, the FRET pairs are gQDs/BHQ1 (donor/acceptor) and rQDs/BHQ2 (donor/acceptor). Upon introduction of labeled targets, the hybridization event brings the acceptors (BHQ1 and BHQ2) in close proximity to the QDs, which allows the BHQs to absorb a portion of the QD emission via the FRET process. This absorption of energy reduces or quenches the QD emission. This serves as the analytical signal to be detected. A higher target concentration implies more BHQs are present, resulting in a greater reduction in the overall observable QD emission signals.

## 4. Detection Using CMOS Technology

The aforementioned challenges call for a solution with reduced platform complexity, size, and cost. This can be achieved using the CMOS technology. CMOS offers many advantages such as low cost, high integration density, and signal processing versatility.

The need for portability can be met with microsystem integration. Integrated circuit based DNA detection platforms have a great potential for point-of-care diagnostic applications because they can be integrated with other technologies to construct compact, self-contained sensing platforms. For example, it is envisioned that biochemical sample preparation can be performed using microfluidic channels with integrated pumps and valves; on-chip solid-state transducers (e.g., CMOS photodiodes) can be used to detect specific DNA sequences in an analyte; and microelectronic integrated circuits can amplify and condition the transducer output signal, convert this information to a digital format, process it in order to extract relevant biochemical data, and then transmit or display these data externally [12,18]. In the following, three key CMOS capabilities are discussed, namely time-resolved, contact, and multispectral imaging.

### 4.1. Time-Resolved Fluorescence Detection

Single-photon avalanche diodes (SPADs) measure the arrival time of individual photons. SPADs in CMOS are becoming increasingly interesting devices for timing applications not only for fluorescence lifetime imaging but also in positron emission tomography and time of flight mass spectroscopy. The CMOS allows integration of functionalities like time-to-digital converters within the same pixel and the manufacturing of large format arrays. The subsection below reviews recently reported work first in architecture and modeling, then in system implementations, and lastly in mitigation of specific issues such as pile-up, dead time, and jitter.

In [32], design issues and challenges of SPAD implementation in CMOS is reviewed on the device, circuit, and system levels. Issues of scalability, miniaturization and performance trade-offs involved in designing SPAD imaging systems are investigated. Design considerations, research challenges, and future directions for CMOS SPAD image sensors are highlighted and addressed.

A configurable model of SPAD array photodetectors with intelligent control and active quenching is presented in [33]. In this model, individual components can be simulated independently and subsequently linked to provide the overall detector response. The model enables the simulation of the entire detector and analysis of performance, including photon detection efficiency acquisition, timing, and energy resolution. It can be used to optimize detector performance for applications not limited to time-resolved spectroscopy but also positron emission tomography (PET). The simulator consists of multiple configurable and interchangeable modules to model the array geometry as well as physical and optical characteristics based on physical models and statistical equations. Readout electronics are also simulated in an algorithmic form. Different methods to extract information from the digital output signal are investigated. The simulator enables the development of new algorithms to extract information from the fluorescence analysis. Simulation results for photon detection efficiency, energy resolution and timing resolution are reported, showing the functionality of the simulator.

The design of high-speed, low-light-detection pixels using CMOS SPAD is described in [34]. This pixel is suitable for fluorescence lifetime imaging (FLIM) used for drug discovery and/or minimally-invasive optical biopsy. In order to achieve high-speed imaging using single-photon detection, a detector with a very low dead-time is needed. The SPAD discussed in this work uses a mainstream deep-submicron CMOS process to achieve ultrahigh-speed operation and high pixel fill-factor, with in-pixel active quench and reset circuits. The paper also presents an innovative approach for reducing the dead time of the detector and a technique that achieves simultaneous high-speed image acquisition by all the pixels of an array in parallel.

To enable imaging, SPADs can be readily tiled into an array in CMOS. In [35], a CMOS imager consists of 32 × 32 smart pixels, each able to detect single photons in the 300–900 nm wavelength range. The pixels can perform both photon-counting and photon-timing operations on very fast optical events with low intensities. In photon-counting mode, the imager provides photon number (*i.e*., intensity) resolved videos at 100k frames/s. In photon-timing, the imager provides photon arrival times with 312 ps resolution. The imager is fabricated in a cost-effective 0.35 μm CMOS technology. Each pixel consists of a SPAD with 30 μm photoactive diameter, coupled to an in-pixel 10-bit time-to-digital converter (TDC) with 320-ns full-scale range, an INL of 10% least significant bit (LSB) and a DNL of 2% LSB. The chip operates in global shutter mode, with a 10 μs full frame time and a 1-ns conversion time. The reconfigurable imager design enables a broad set of applications related to time-resolved spectroscopy, such as fluorescence lifetime imaging, molecular imaging, and diffusive optical tomography.

Time-correlated single-photon counting (TCSPC) imagers have become very capable due to high level of integration enabled by CMOS. A fully-integrated SPAD and TDC array for high-speed FLIM in standard 130 nm CMOS is presented in [36]. This imager consists of an array of 64 × 64 SPADs each with an independent TDC for performing per-pixel TCSPC. The TDCs use a delay-locked-loop-based architecture, achieving a 62.5 ps resolution with a 64 ns range. A data compression datapath transfers TDC data to off-chip buffers, which can support a data rate of up to 42 Gbps. These features, combined with a system implementation that leverages a 4× PCI express interface, allow for demonstrated 100 frames/s FLIM imaging rates.

A high dynamic range is needed to fully satisfy many SPAD applications. In [37], a CMOS SPAD time-domain imager achieving a high dynamic range is presented. Operating the SPADs in Geiger mode, the imager also demonstrated high sensitivity, high-speed operation.

A smart pixel based on the SPAD for TCSPC applications, fabricated in a commercial 0.35 μm CMOS technology, is presented in [38]. The large CMOS detector (30 μm active area diameter) shows very low noise (12 counts per second at room temperature at 5V excess bias) and high efficiency in a wide wavelength range (about 50% at 410 nm and 5% at 800 nm). The analog front-end electronics senses and quenches the avalanche, thus leading to an almost negligible after-pulsing effect. The in-pixel 10-bit TDC provides 312 ps resolution and 320 ns full-scale range.

Noise reduction techniques can be built into the SPAD imager. The design of an active, integrated CMOS sensor array for fluorescence applications which enables time-gated, time-resolved fluorescence spectroscopy is presented in [39]. The 64 × 64 array is sensitive to photon densities as low as 8.8 × 10^6^ photons/cm^2^ with 64-point averaging. Through a differential design, the pixel has a measured impulse response of 800 ps. The imager is suitable for both active microarrays and high-frame-rate imagers for fluorescence lifetime imaging microscopy.

Pulse pile-up is one of the most fundamental limitations of time-resolved systems. Pulse pile-up mitigation has been integrated in CMOS SPAD imagers. In [40], a 64 × 64 pixel CMOS SPAD imager for time-resolved fluorescence detection features actively quenched and reset pixels is presented. The imager allows gated detection to eliminate pile-up nonlinearities common to most TCSPC approaches. Reset timing information is collected using an on-chip TDC based on a counter and a supply-regulated delay-locked loop.

The powerful technique of FRET can be combined with time-resolved techniques. In [41], an active oligonucleotide microarray platform for time-resolved FRET (TR-FRET) assays is presented. In these assays, immobilized probe is labeled with a donor fluorophore and analyte target is labeled with a fluorescence quencher. Changes in the fluorescence decay lifetime of the donor are measured to determine the extent of hybridization. The work demonstrates that TR-FRET assays have reduced sensitivity to variances in probe surface density compared with standard fluorescence-based microarray assays. Use of an active array substrate, fabricated in standard CMOS, provides the additional benefits of reduced system complexity and cost. The array consists of 4096 SPAD pixels and features an on-chip TDC. The presented system demonstrates the measurement of a DNA target concentration series using TR-FRET with semiconductor QD donors.

Aside from demonstrating system-level prototypes, a number of works focus on improving specific aspects of the time-resolved system, such as time-resolution variation. A 128-channel column-parallel two-stage TDC utilizing a time difference amplifier (TDA) is proposed in [42]. Measurement results from a 0.35 μm CMOS implementation are presented. The first stage operates as a coarse TDC, the time residue is amplified by a TDA, then converted by the second-stage TDC. As the gain of the TDA is adjusted from 8.5 to 20.4, the time resolution of the TDC can be tuned from 21.4 to 8.9 ps. The time resolution variation due to process-voltage-temperature (PVT) effects is ±5.8% without calibration when the time resolution is 12.9 ps. A calibration method is also proposed to compensate for LSB changes due to the power supply fluctuation and temperature variation.

Aside from mitigation of variation, non-ideal characteristics such as dead time have to be taken into account in order to correctly interpret SPAD measurements. In [43], a model for dead time in real SPADs where reset is generated off-pixel is derived and tested. A Monte Carlo simulation is implemented to compare with experimental results obtained from a 180 nm CMOS prototype. Using a fitting method, higher values of the photon detection efficiency (PDE) can be extracted than with a simple linear fit. It is found that accurate predictions of the true count rate are possible over a control range of 0.25–1.0 MHz.

Aside from a short dead time, a low detector jitter is required in many SPAD applications. A method for significantly reducing SPAD jitter using an area-efficient shallow-trench-isolation guard ring is presented in [44]. The structure prevents lateral drift and diffusion of charge carriers, resulting in improved timing resolution. Experimental results of a 0.18 μm CMOS prototype are presented. The timing resolution of the SPAD is 27 ps full-width at half-maximum. The diffusion tail exhibits only 96 ps full-width at hundredth-maximum.

### 4.2. Contact Imaging

**Figure 3.**
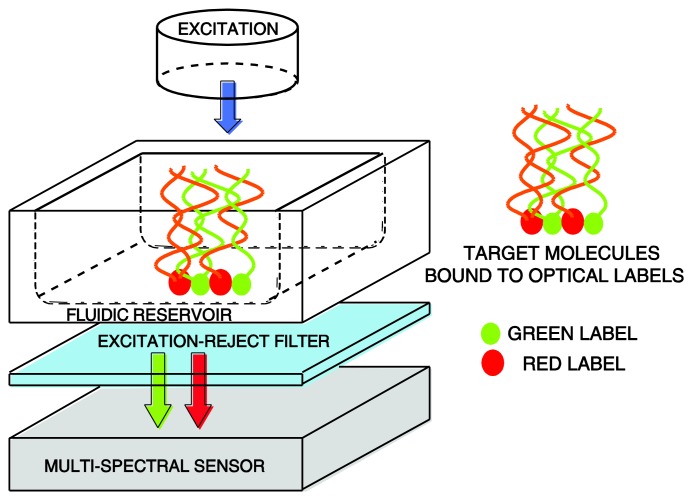
CMOS fluorescent contact imaging microsystem schematic.

**Figure 4.**
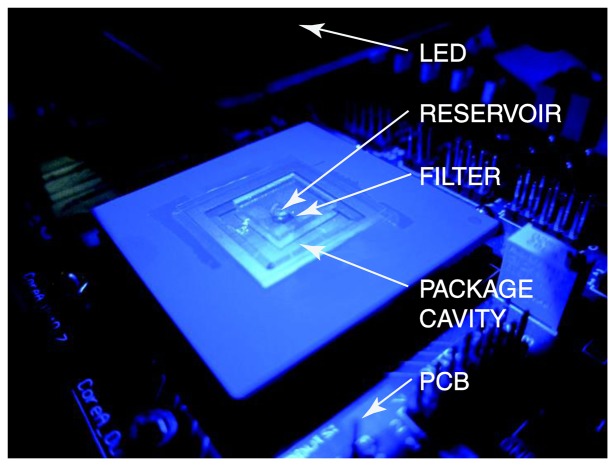
CMOS fluorescent contact imaging microsystem implementation.

To enable small-form-factor, a contact fluorescent detection microsystem can be used to excite the fluorescent markers and quantitatively detect their emission, which is representative of the target concentration. Reference [2] reported such contact detection microsystem. The schematic and implementation of the microsystem are depicted in Figures 3 and 4, respectively.

The microsystem consists of a blue light-emitting diode (LED) for excitation, a collimator to ensure the excitation light reaching the sample and the filter has a normal angle of incidence, an optical emission filter, a fluidic structure for holding the sample solution, and for photo detection, a CMOS multispectral sensor. The optical filter chosen for the microsystem is a discrete thin-film interference filter for attenuating the excitation light from the Philips Luxeon K2 450 nm blue LED excitation (FWHM = 20 nm). The long-pass interference filter has a cut-off wavelength of 510 ± 2 nm. The cut-off wavelength is chosen to transmit the emitted light with the peak emission wavelengths of 520 nm and 620 nm, respectively.

In [45], the design, fabrication, and verification of a novel CMOS imager-based contact imaging system are presented. Fluorescent images are acquired from live neurons by monitoring calcium changes with Fura-2 dye. The device consists of a removable absorption filter interfaced with a CMOS imaging sensor and an external DG-4 lamp for excitation. Fura-2 loaded Lymnaea stagnalis neurons are stimulated with dual excitation wavelengths of 340 and 380 nm. The image sensor detects 510-nm emission, verifying that the system is capable of detecting intracellular calcium changes in Fura-2 loaded neurons. Further, the sensor also enabled viewing of multiple neurons over a large surface area simultaneously, an option that is not readily available in conventional light microscopy.

In [10], a hybrid CMOS/thin-film microsystem for DNA fluorescence contact imaging is presented. The microsystem integrates a high-performance optical interference filter and a 128 × 128 pixel active pixel sensor fabricated in a standard 0.35-μm CMOS technology. The thin-film filter has an optical density greater than 6.0 at the wavelength of interest, providing adequate excitation rejection to the 532 nm solid-state laser. Microsystem performance is experimentally validated by imaging spots of Cyanine-3 fluorophore, conventionally used in DNA detection. The emission intensity as a function of fluorophore concentration is measured with an estimated sensitivity of 5000 fluorophore/μm^2^. An 8k-spot human DNA microarray has been imaged with the sensor prototype.

Miniaturized fiber optics can be used to enhance optical performance. The work reported in [4] describes a bioluminescence detection lab-on-chip consisting of a fiber-optic faceplate with immobilized luminescent reporters/probes that is directly coupled to an optical detection and processing CMOS system-on-chip (SoC) fabricated in a 0.18-μm process. The lab-on-chip is customized for such applications as determining gene expression using reporter gene assays, determining intracellular ATP, and sequencing DNA. The CMOS detection SoC integrates an 8 × 16 pixel array having the same pitch as the assay site array, a 128-channel 13-bit ADC, a column-level digital signal processor (DSP), and is fabricated in a 0.18 μm image sensor process. The chip is capable of detecting emission rates below 10^−6^ lux over 30 s of integration time at room temperature. In addition to directly coupling the assay sites to the photodetectors, this low light detection is achieved by a number of techniques, including the use of very low dark current photodetectors, low-noise differential circuits, high-resolution analog-to-digital conversion, background subtraction, correlated multiple sampling, and multiple digitizations and averaging to reduce read noise. Electrical and optical characterization results as well as preliminary biological testing results are reported.

Circuit techniques are often used to enhance signal-to-noise performance. A 132 × 124 high sensitivity imager array with a 20.1-μm pixel pitch fabricated in a standard 0.5-μm CMOS process is presented in [20]. The chip incorporates n-well/p-substrate photodiodes, capacitive transimpedance amplifier (CTIA) based in-pixel amplification, pixel scanners, and delta differencing circuits. The 5-transistor all-nMOS transistor pixel interfaces with peripheral pMOS transistors to form a column-parallel CTIA. At 70 frames/s, the array has a minimum detectable signal of 4 nW/cm^2^ at a wavelength of 450 nm while consuming 718 A from a 3.3 V supply. The peak signal-to-noise ratio (SNR) is 44 dB at an incident intensity of 1 W/cm^2^. Implementing 4 × 4 binning allowed the frame rate to be increased to 675 frames/s. Alternately, sensitivity can be increased to detect 0.8 nW/cm^2^ at 70 frames/s. The chip is used to image single-cell fluorescence at 28 frames/s with an average SNR of 32 dB.

The compactness enabled by contact imaging is best showcased in handheld applications. The design, fabrication, and characterization of a class of simple handheld fluorometers are described in [24]. The devices consist of a sensor along with an integrated optical filter packaged in a handheld format. The sensor is a differential active pixel sensor with in-pixel correlated double sampling fabricated in a 0.5-μm 2-poly 3-metal CMOS process and has a readout noise of 175.3 μV, reset noise of 360 μV, dynamic range of 59 dB, and conversion gain of 530 nV/e^−^. The emission filter is a high rejection chromophore embedded in a polymer film which is cast onto the chip. The work shows results of bioassays with two different single color fluorometers constructed by using the chromophores 2-(2′-hydroxy 5′-methylphenyl) benzotriazole and Sudan II with long-pass wavelengths of 400 nm and 540 nm, respectively. The bioassays measure metabolic activity and viability of biological cells, which are useful for cytotoxicity and pathogen detection applications.

A major challenge for contact imaging is that the sample and photodetector are separated by the emission filter. In other words, the sample-to-detector distance, which is determines the spatial resolution, depends on the filter thickness. For example, the interference filter used in [2] is fabricated using a 60-layer coating of Nb_2_O_5_ and SiO_2_. The coating is deposited onto a microsheet of fused silica substrate by vapor deposition, resulting in a total filter thickness of approximately 100 μm. The constraint on filter thickness may force the designer to tradeoff excitation rejection performance for spatial resolution. In [46], a wide dynamic range CMOS contact imager is proposed as a solution by tolerating a small amount of stray excitation without sensor saturation. The work also presents a quantization power optimization scheme where photon shot-noise is taken into consideration. An analysis is presented for the wide dynamic range asynchronous self-reset with residue readout architecture. An implementation of this architecture is presented where the (coarse) asynchronous self-reset operation and (fine) residue analog-to-digital conversions are performed with separate in-pixel and off-pixel circuits, respectively. A prototype, fabricated in a standard 0.35 μm CMOS process, achieves a measured dynamic range of 82 dB with a peak SNR of 46.2 dB under broadband illumination. The prototype also incorporated a microfluidic device, demonstrating integrated sample handling.

### 4.3. Spectral Multiplexing

Differentiation between fluorescent emission wavelengths has been conventionally achieved by using a set of optical bandpass filters to select different parts of the emission spectrum. The optics involved is bulky and expensive. To circumvent this problem, alternative spectral methods have been investigated.

Methods based on diffraction grating (the splitting of light) [47] and Fabry-Perot etalon (tuned resonance cavity) [48] generally offer high spectral resolution, but require micromachining and post-processing such as wafer polishing and wafer bonding. Eliminating the need for sophisticated optics and post-processing is the ultimate remedy to the high design complexity and fabrication cost [49]. Subsequently, a recently reported CMOS-based multi-spectral technique is described in detail.

In [2,5], a CMOS multispectral sensor is proposed to detect and differentiate the multi-color emission, eliminating the need to mechanically swap optical filters. As a result, the complexity of the microsystem is significantly reduced by the integration of the CMOS sensor. The CMOS multispectral sensor is based on the color photogate (CPG) structure and operates as follows. When multiple wavelengths of light are incident simultaneously, the intensities at these wavelengths can be determined by measurements from multiple photo detectors with unique spectral responses. For example, for a two-wavelength input, the photo currents *I_1_* and *I_2_* measured by two photo detectors can be related to the input intensities *ϕ_1_* and *ϕ_2_*, at wavelengths *λ_1_* and *λ_2_* respectively, by

(1) $$I_{1}=k_{11}\phi_{1}+k_{12}\phi_{2}$$

(2) $$I_{2}=k_{21}\phi_{1}+k_{22}\phi_{2}$$

where the *k*-coefficients capture the responsivity of the detectors at different wavelengths and can be obtained empirically. The input intensities *ϕ_1_* and *ϕ_2_* can be obtained by solving the system of equations, provided that the detectors have unique spectral responses (*i.e*., equations are linearly independent). This model can be extended to a finite set of *N* wavelengths. To determine the intensity of an input spectrum to a resolution of *N* distinct wavelengths, *N* measurements are required from the *N* detectors (one measurement per detector), with each detector having a unique spectral response.

To create the equivalent of multiple photo detectors with unique spectral responses, the CPG has been realized in 0.35 μm CMOS. The CPG is in fact implemented as a *p*-channel metal-oxide-semiconductor field-effect transistor (MOSFET) with its source and drain terminals connected. It demonstrates that a standard CMOS transistor can in fact be used as a multi-spectral detector. Each 175 μm × 175 μm pixel is tiled to form an array for imaging. Each pixel consists of a 50 μm × 50 μm CPG and an ADC for digital readout.

**Figure 5.**
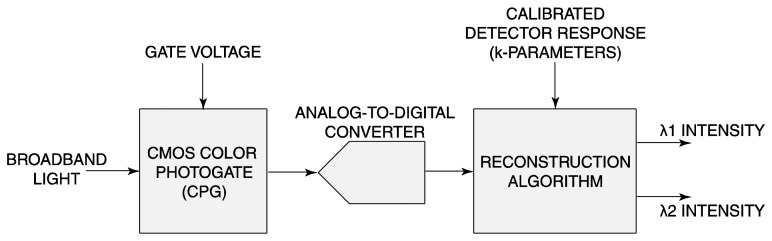
System diagram of the CMOS multispectral sensor.

Figure 5 depicts the overall CPG-based sensor architecture. To resolve *N* colors, *N* CPG responses at *N* different values of CPG gate voltages are passed through the ADC for digitization. The reconstruction algorithm that solves for the input spectrum *ϕ* in Equations (1) and (2) is implemented in software. The microsystem has been applied to the simultaneous detection of two DNA targets, with results comparable to those obtained from a commercial fluorescence microscope.

## 5. Conclusions

CMOS implementation of three fluorescence detection techniques, specifically time-resolved, contact, and multispectral imaging are discussed. State-of-the-art CMOS realizations provide ample evidence that requirements for biosensing are well supported by the high integration density, low fabrication cost, and versatile signal processing capabilities of the technology. CMOS has demonstrated itself to be an enabling technology for miniaturized fluorescence detection microsystems, suitable for a new breed of portable, point-of-care diagnostic applications.
